# Providing Oral Health Education to Adolescents with Peer-Assisted Learning

**Published:** 2020-08-18

**Authors:** R. Constance Wiener, Kimberly Bailey, Amelia Adcock, Scott Young, Summer Kuhn, Catherine Morton

**Affiliations:** 1 Dental Practice and Rural Health, West Virginia University, USA, 304 581-1960; 2 Department of Surgery, West Virginia University, USA, 304 598-1106; 3 Department of Neurology, West Virginia University, USA, 598-6127; 4 Department of Restorative Dentistry, West Virginia University, USA 304 293-1129; 5 Health Sciences and Technology Academy, West Virginia University, USA; 6 Health Sciences and Technology Academy, West Virginia University, USA

**Keywords:** peer-assisted learning, near-peer, oral health, literacy

## Abstract

There is a need to increase oral health knowledge, attitudes and behaviors in children to improve oral health. This research involves peer-assisted learning to determine if high school students can influence rural middle school students’ oral health. The study sample consisted of middle school students. After completing pre-test, they were assigned to receive 1) didactic peer-assisted learning with professionally supervised and educated high school students (members of an after-school pipeline program for science, technology, engineering, mathematics, and health science); or, 2) teacher provided handouts/activity sheets. Both groups then completed a post test. The results of the Mann-Whitney U Tests showed that brushing and flossing failed to reach significant improvements between the pre-test and post-test for the handouts/activity sheets group (brushing, P=0.391; flossing, P=0.459). There was improvement within that group for oral health knowledge (P<.001). Brushing, flossing and oral health knowledge failed to reach significant improvement between the pre-test and post-test for the peer-assisted learning group (brushing, P=0.760; flossing, P=0.707; oral health knowledge, P= 0.154). In terms of oral health knowledge, there was no difference between the scores of the two groups on the pre-test (P-value = 0.980) nor on the post-test (P-value= 0.237). Near-peer assisted learning for oral hygiene knowledge, attitudes, and behaviors had similar outcomes as teacher provided handouts and activity sheets in a middle school setting.

## Introduction

Developing the habit of daily oral infection control through brushing and flossing is important to maintain personal oral health. Nevertheless, in one report, nearly 25% of children were not developing appropriate brushing and flossing habits [1]. It is known that middle school children benefit academically from cross-age peer-assisted learning in many academic areas and similarly mentors also often benefit from this relationship [2,3]. Peer-assisted learning has been defined to include peer-to-peer or near-pear relationships. Near-peers are two participants who are at least one academic year apart [4]. The teaching relationship can take the form of mentoring (if 1–2 students are involved); tutoring (if 3–10 students are involved); or may be didactic (if more than 10 students are involved) [4].

Schools are a natural setting for peer-assisted learning [5]. Previous researchers have reported that students involved in peer-assisted learning become less intimidated (have psychological safety) and more comfortable (have social support) than students who do not. They also gain understanding and perspectives from the peer who may have had similar learning challenges. The near-peer has an opportunity to reflect upon previous knowledge, gain teaching skills, interact with students and teachers, gain respect, and have a rewarding experience in helping younger students [6]. Peer-assisted learning has been successful in several areas.

It has been also reported to be effective with children 7–13 years in terms of behavior improvement (violence prevention) [7]. The use of cross-age peer-assisted learning for daily oral infection control knowledge and behavior has not been previously researched with middle school students. The purpose of this study was to determine if a peer-assisted learning approach would impact oral health knowledge/attitudes/beliefs and skills for middle school children.

## Materials and Methods

This study received West Virginia University Institutional Review Board approval (IRB protocol number 1803018663).

### Procedure

The researchers contacted Health Science and Technology Academy (HSTA) high school students to help with the research as near peers for the middle school children. HSTA students are interested in careers to help the medically under-served and they have presented healthcare outreach sessions to adults throughout their rural communities [9–12]. The researchers used the Bransford “How People Learn Framework” learning theory as their conceptual framework for the study. In the theory there are four lenses to facilitate learning: 1) knowledge-centered; 2) learner-centered; 3) assessment-centered; and 4) community environments [13]. The knowledge-centered aspect of the framework was used to organize information for the middle school children’s level of understanding. The packaging of information included efficient use of media (traditional PowerPoint slides with bulleted highlights; pencil and paper work sheets, and the innovative use of technology such as Kahoot!® and other platforms that increased interaction while focusing upon content). Learning was designed to be assessment-centered for this project as assessments offer opportunities to reconsider aspects of the program and revise the curricula to enhance learning based upon the feedback provided by the students. Learning was also designed to be community-oriented—in this case the students in the classroom were the community. Near-peers were non-threatening, empathetic, and able to help younger students feel less fearful of speaking, thereby strengthening lines of communication. The theory’s emphasis on providing usable knowledge was also a rationale for preferring the Bransford theory for developing the program. The communication emphasis, and active inquiry-based approach were important to uncover students’ false beliefs and naïve understandings [13] about oral health.

### Study sample

245 middle school students from voluntarily enlisted schools in West Virginia were invited to participate in a cross-age mentoring project with HSTA high school student oral health near peers. Middle school students provided assent and parents provided consent for the research.

### Baseline assessment and group assignments

A pre-test was created by the study team with input and review of the HSTA students. It was the baseline assessment of oral health knowledge/attitudes/beliefs and had a self-report of brushing and flossing (oral health behaviors). Community research associates and classroom teachers discussed the assignment of the schools to either an intervention school or a control school. The criteria for the assignments were that the administration of the program would have the least class time disturbance (a convenience sampling). Therefore, a cluster design was employed. The middle school students in the intervention schools had the HSTA student near-peers intervention. The middle school students in the control schools received oral health handouts.

### Near-Peer Education and Control classrooms

The HSTA students provided the in-person, near-peer education. The topics were: daily oral infection control; oral and systemic effects of tobacco use and vaping; oral health and nutrition; and, trauma and oral injuries. The interventions included inquiry-based activities. The HSTA high school students met with West Virginia University Health Sciences faculty members via four teleconferences to review the objectives and activities of the research prior to the sessions with the middle school students. Each of the sessions with the West Virginia University faculty members and HSTA high school students was approximately one hour in length. The HSTA high school students received additional instruction from their community research associates. The HSTA students and community research associates revised activities to be consistent with students’ existing knowledge and understanding of daily oral infection control. Lesson plans were created to engage middle school students using technology such as Kahoot!,® PowerPoint® slides, as well as with handouts, dental models, and volunteer-led demonstrations.

### Variables Examined

The data for the research were collected and managed with the REDCap (Research Electronic Data Capture) electronic data capture tools hosted at West Virginia University. REDCap is a secure, web-based application that provides 1) a data entry interface; 2) audit trails 3) data exporting; and 4) data importing [14]. Data relating to self-reported oral health behavior were measured as the number of times a day a middle school student brushed; flossed; drank milk; or drank soda. The data related to oral health attitudes were measured as the importance of having white teeth, healthy teeth, maintaining teeth over one’s lifetime, and the selection of treatment choices. Additionally the responses included the perception of the importance of healthy teeth to one’s family and friends, the perception of having enough time to brush and floss, and the perception of having a good toothbrush to use. Oral health knowledge gained was measured by the change in correct responses concerning the four sessions with the HSTA near-peers (for the intervention group).

The discrete tools used are presented in Tables 1 and 2.

### Statistical analysis

The pre-test and post-test had 19 knowledge-based statements to which middle school students were to agree or disagree. The responses on the pre-test and post-test were dichotomized to “correct” and “incorrect“ responses. A neutral response was categorized as incorrect. A score of zero was assigned to incorrect answers and a score of 1 was assigned to correct responses. The number of correct responses, means and standard deviations on each test was determined. Daily oral infection control and other oral health behaviors and attitudes were collected. Results were compiled using descriptive statistics. Mann-Whitney U Tests were used to compare the groups. Data analysis was conducted with IBM SPSS® Version 25 (Carey, NC). The P-value was set at <.05.

## Results & Discussions

There were 245 middle school students (52.3% male, n=124) who participated in the pre-test. Of these students, 94.6% were in seventh grade, and their mean age was 13.20 years (SD, 0.75 years). There were 58.5% reporting dental insurance coverage. Seventeen students (7.5%) reported one urgent dental visit within the previous year, and 13 (13.2%) reported more than one urgent visit to his or her dentist within the previous year.

There were 28 students (12.7%) reporting no dental visits within the previous year; 39 (17.6%) reporting one dental visit within the previous year; and, 154 (69.7%) reporting two or more dental visits within the previous year. Eighty-three students (38.2%) reported at least one dental restoration; and, 51 (21.9%) reported having teeth that hurt. There were 174 students in schools assigned as controls and 71 students in schools assigned to have HSTA students.

Before the study, the control and intervention groups were similar on oral health behaviors, oral health attitudes, and oral health knowledge (Tables 1 and 2). There was one behavior which was different between the groups, the number of glasses of milk consumed, which was slightly higher in the intervention group. There were no changes in improvement in daily oral infection control behavior with respect to brushing for either the control group (p=0.391) or intervention group (p=0.760). There were also no changes in improvement in daily oral infection control behavior with respect to flossing for either the control group (p=0.459) or intervention group (p=0.707).

## Discussion

In similar studies involving science, technology, electronics, mathematics [16–18] and reading [19] near-peer assisted learning was reported to be helpful. The results of this study do not support those results. It is possible that the topic itself was not amenable to near-peer intervention. Dental terminology is complex. Brushing and flossing skills and habit development require commitment as students are being asked to brush twice daily and floss daily for the rest of their lives. Even adults have difficulty with dental knowledge and practices.

In a qualitative study evaluating peer-assisted learning (specifically mentoring), several challenges were noted that may also apply to this current study. Small groups were found to be more conducive to learning than larger groups [20]. Near-peers often are very enthusiastic about their topic, but it is irrelevant if the learner does not share the enthusiasm [20]. In this research study, there were four structured sessions with the middle school students. It is possible that there was not sufficient time for a strong near-peer relationship to develop to facilitate learning, and skill/habit-development.

### Limitations

One criticism of peer-assisted learning is the inadequacy of preparation during peer training and inadequate subsequent monitoring of the accuracy of implementation [21]. A strong point of this research was the intensive HSTA student training by both the WVU faculty members and the community research associates. Preparation involved the collaboration and development of materials for use by the HSTA students by the WVU faculty members, community research associate researchers, and the HSTA students. Additionally, there was supervision of the HSTA students by the community research associates during their interactions with the middle school students to ensure fidelity of the information provided.

Start-up costs are also limitations noted in another research [21]. Without adequate funding, there is often a limitation of the effectiveness of peer-assisted learning; however, the university researchers self-funded the handouts and activity sheets and the community research associate researchers self-funded dental models for demonstrating proper brushing and flossing. Other such programs would have financial considerations to determine implementation.

In a meta-analysis of peer-assisted learning with elementary students, there was a positive increase in achievement [22]. However, perhaps such interventions are more effective with younger, urban, low-income, minority students [22]. The current research involves older (middle school) students who are rural and primarily not minority students.

The results of this research indicate a greater need for oral health education in Middle School curricula. Future research could potentially involve a mixed approach with both dental access to dental healthcare professionals and longer interventions with HSTA students. Many of the techniques used by the near-peers were innovative and engaging. These also should be evaluated in future research to determine the effectiveness of technological features used by near-peers and younger students to teach and learn about oral health.

## Conclusion

In terms of oral health knowledge, there was no difference between the scores of the two groups on the pre-test (P-value = 0.980) nor on the post-test (P-value= 0.237). Near-peer assisted learning for oral hygiene knowledge, attitudes, and behaviors had similar outcomes as teacher provided handouts and activity sheets in a middle school setting.

## Figures and Tables

**Table 1:** Oral Health Behaviors and Attitudes

	**Pre-test Mean (SD) 245**			**Post-test Mean (SD) 169**		
	**n= 174 Control**	**n=71 Intervention**	***P*-value^1^**	**n= 151 Control**	**n= 18 Intervention**	***P*-value^1^**
**Oral health Behaviors**						
Mean number of times brushing per week	10.11 (5.49)	10.46 (4.74)	.406	11.39 (6.63)	9.29 (5.11)	.297
Mean number of times flossing per week	3.33 (4.07)	3.79 (4.35)	.784	3.40 (4.24)	3.78 (3.96)	.551
Mean number of sodas, sugar sweetened beverages yesterday	1.86 (2.28)	1.73 (1.83)	.975	1.17 (1.28)	1.65 (1.46)	.157
Mean number of glasses of milk consumed yesterday	0.87 (1.08)	0.97 (1.27)	.020	0.87 (1.18)	1.53 (1.42)	.047
	**Pre-test Mean Likert Scale value^2^ (SD)**			**Post-test Mean Likert Scale value^2^ (SD)**		
	**Control**	**Intervention**	***P*-value^3^**	**Control**	**Intervention**	***P*-value^3^**
**Oral Health attitudes^1^**						
Importance to have white teeth	1.96 (.81)	1.83 (.73)	.215	1.82 (.84)	1.88 (.70)	.598
Importance to keep teeth for a lifetime	1.49 (.66)	1.45 (.61)	.185	1.43 (.62)	1.44 (.63)	.931
Choice of root canal therapy over extraction	2.65 (1.03)	2.36 (.91)	.165	2.46 (1.08)	2.06 (1.09)	.153
It is important to me to have healthy teeth.	1.51 (.70)	1.46 (.61)	.760	1.45 (.62)	1.50 (.62)	.677
It is important to my family for me to have healthy teeth.	1.69 (.89)	1.65 (.68)	.456	1.62 (.80)	1.61 (.70)	.858
My friends’ value healthy teeth	2.43 (.89)	2.40 (.92)	.220	2.23 (.96)	2.47 (1.13)	.436
I have enough time each day for me to brush and floss	1.89 (.98)	1.91 (1.06)	**.047**	1.83 (.91)	1.72 (.83)	.672
I have a good toothbrush	1.85 (.84)	1.55 (.63)	.529	1.71 (.69)	1.56 (.62)	.389

Abbreviations: n, number; SD, standard deviation

1 *P*-value for comparison of responses in pre-test between control and intervention using Independent Samples Mann-Whitney U Test.

2 The means are based on the Likert scale where: 1=strongly agree; 2=agree; 3=neutral; 4=disagree; and 5= strongly disagree.

3 *P*-value for comparison of responses in post-test between control and intervention using Independent Samples Mann-Whitney U Test.

The p-value for improvement in the control group from the pre-test to the post-test was <.001 for soda consumption using Independent Samples Mann-Whitney U Test. All other behaviors and attitudes in the control group and the intervention group had p-values >.05 from the pre-test to the post-test.

**Table 2 :** Oral Health Knowledge, Pre-test and Post-test

	Correct Response	Pre-test		Post-test	
		n= 174 Control n, %	n=71 Intervention n, %	n= 151 Control n, %	n= 18 Intervention n, %
DAILY ORAL INFECTION CONTROL					
A medium toothbrush is better than a soft or hard toothbrush.	False	89, 51.1%	43, 60.6%	61, 40.1%	13, 72.2%
A person should brush his or her teeth for at least 1 minute.	False	27, 15.5%	12, 16.9%	46, 30.3%	1, 5.6%
Fluoride is added to water to disinfect it.	True	25, 14.5%	8, 11.3%	27, 17.8%	0
Tooth decay bacteria can pass among people when sharing the same spoon, fork, or glass.	True	76, 43.7%	30, 42.3%	107, 70.4%	9, 50.0%
A teenager should use a pea-sized amount of toothpaste.	False	71, 40.8%	31, 43.7%	106, 69.7%	10, 55.6%
The American Dental Association recommends brushing 2 times a day.	True	145, 83.3%	62, 87.3%	137, 90.1%	17, 94.4%
A person should use at least 36 inches of floss to properly floss.	False	49, 28,2%	12, 16.9%	49, 32.2%	2, 11.1%
The American Dental Association recommends brushing 2 times a day.	True	132, 75.9%	55, 77.5%	114, 75.0%	13, 72.2%
Gingivitis is a reversible disease of the tissue around teeth.	True	63, 36.3%	31, 43.7%	89, 58.6%	13, 72.2%
Periodontal disease involves the bone around the teeth.	True	59, 33.9%	23, 32.4%	88, 57.9%	9, 50.0%
TOBACCO AND ORAL HEALTH					
Smoking is a risk factor for gum disease.	True	153, 87.9%	62, 87.3%	136, 89.5%	17, 94.4%
Chewing tobacco is a risk factor for oral cancer.	True	151, 86.8%	67, 94.4%	137, 90.1%	17, 94.4%
More than ½ (68.7%) of first time smokers become daily smokers.	True	126, 72.4%	52, 73.2%	116, 76.3%	13, 72.2%
INJURY AND ORAL TRAUMA					
Biking helmets do NOT protect the teeth.	True	104, 59.8%	45, 63.4%	95, 62.5%	13, 72.2%
Car seat belts are important in helping to prevent facial injuries.	True	134, 77.0%	55, 77.5%	122, 80.3%	14, 77.8%
In football, a player should lead with his/her head to prevent facial	False	51, 29.3%	18, 25.4%	67, 44.1%	8, 44.4%
NUTRITION, ORAL, AND SYSTEMIC HEALTH					
It is better for your teeth to sip a soda for 1 hour rather than to drink it all at once.	False	49, 28.2%	16, 22.5%	58, 38.2%	3, 16.7%
Most people in the U.S. have a diet with 10% or less sugar.	False	40, 23.0%	15, 21.1%	41, 27.0%	4, 22.2%
If a nutrition label has 2 1/2 servings in the package and there is 20 g or sugar per Serving, a person would eat 30 g of sugar if he or she ate everything in the package.	False	41, 23.6%	17, 23.9%	40, 26.5%	2, 11.1%
	*P*-value between groups^1^ .980			*P*-value between groups^2^ .237	
**Overall mean percentage correct**	**48.0%**		**48.0%**	**56.8%**	**52.1%**
**Mean number correct (SD)**	**9.1 (3.09)**		**9.2 (3.09)**	**10.8 (3.24)**	**9.89 (3.14)**

Abbreviations: n, number; SD, standard deviation

1 p-value for comparison of overall score in pre-test between control and intervention using Independent Samples Mann-Whitney U Test.

2 p-value for comparison of overall score in post-test between control and intervention using Independent Samples Mann-Whitney U Test.

The p-value for improvement in the control group from the pre-test to the post-test was <.001 using Independent Samples Mann-Whitney U Test.

The p-value for improvement in the intervention group from the pre-test to the post test was .154 using Independent Samples Mann-Whitney U Test.
